# Increasing the analysis rate of automatic analysers by subtractively correcting for specimen interaction

**DOI:** 10.1155/S1463924681000382

**Published:** 1981

**Authors:** J. H. T. Bates, A. E. McKinnon, T. A. Walmsley

**Affiliations:** 1 Department of Medicine Christchurch Clinical School of Medicine Christchurch Hospital Christchurch New Zealand; 2 Department of Biochemistry Christchurch Hospital Christchurch New Zealand


### Increasing the analysis rate of automatic analysers by subtractively correcting for specimen interaction

**J. H. T. Bates and A. E. McKinnon**
*Department of Medicine, Christchurch Clinical School of Medicine, Christchurch Hospital, Christchurch, New Zealand.*

**T. A. Walmsley**
*Department of Biochemistry, Christchurch Hospital, Christchurch, New Zealand.*

### Introduction

Much of the routine analysis of biological specimens (eg plasma, urine) in hospital biochemistry laboratories is performed by automatic analysers. These are machines that automatically subject the specimens to a set of physical or chemical conditions and subsequently monitor some function of the specimens (eg photoabsorption at a particular wavelength). An automatic analyser samples each specimen for a set length of time and samples a wash solution between specimens. Throughout the analytical procedure the materials being analysed are transported through considerable lengths of thin tubing as small fluid elements separated by bubbles. Temporary attachment of material to the walls of the tubing causes a small amount of contamination of each fluid element by the contents of the preceding element. When the automatic analyser switches from sampling the wash solution to sampling a specimen the analyser response is an exponential rise instead of a sharp step. Similarly, when the automatic analyser ceases to sample the specimen and begins to sample the wash solution again the machine response is an exponential fall. The response of the automatic analyser to a specimen is a peak (as exemplified by the response to a bicarbonate standard shown in Figure 1) rather than a square pulse.

The operation of automatic analysers is constrained by the length of time for which each specimen must be sampled (which must be sufficient to elicit a measurable response from the analyser) and by the number of specimens that can be analysed in a given time (the throughput of the automatic analyser). In laboratories with high work loads a high automatic analyser throughput is of vital importance. Usually, throughput is increased by decreasing the wash solution sampling time, with the result that the peaks associated with individual specimens partially overlap (Figure 2). Thus, the apparent height of each peak is greater than the true height due to the addition of the tail of the preceding peak (except for the first of a series of peaks). This effect is called specimen interaction [1]. Corrections to the apparent height of each peak can usually be made by the carry-over correction method [1,2], which involves subtracting from the apparent height of the peak the appropriate fraction of the apparent height of the preceding peak. However, this method fails to give accurate corrections to the apparent height of a small peak preceded by a taller one when the wash solution sampling time is very short. The smaller peak appears as a shoulder on the higher peak so that even its apparent height cannot be determined accurately (Figure 2a).

A method of curve regeneration which estimates peak heights from the initial rate of rise of each peak has been used to increase automatic analyser throughput from that possible using the carry-over correction method [3-5]. Because this method effectively differentiates the initial part of each peak it is particularly sensitive to noise in the data.

This paper presents an alternative method of correcting for specimen interaction (the subtractive method) which avoids the disadvantages of the two methods previously described, and offers a significant increase in throughput.

### The subtractive correction method

The basis of the subtractive method for correcting for specimen interaction is that each peak can be represented by a model peak with appropriate vertical scaling. The details of the procedure are as follows.

1. The apparent height of the first peak in a series is taken as its true height. The leading edge and apex of the first peak in a series are unaffected by other peaks, and the automatic analyser is thoroughly flushed with wash solution prior to analysis of the first specimen (consider, for example, the series of five bicarbonate response peaks shown in Figure 2a).
2. A model peak is scaled (by linear least squares) to match the leading edge and apex of the first (left-hand-most) peak. The tail of the first peak is determined (Figure 2b) in this manner.

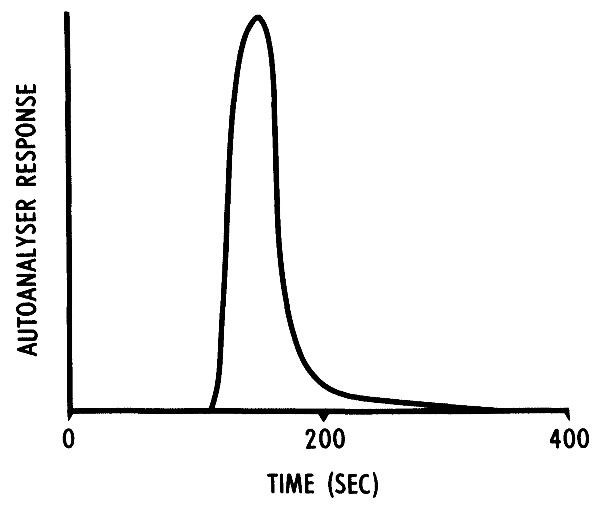

*Figure 1. The peak produced by an automatic analyser after analysis of a bicarbonate standard.*

3. This complete first peak is subtracted from the original data to provide the true height of the next peak (to the right of it) (Figure 2c).

4. This process is repeated from step (2) for all peaks. Note how the subtractive method is able to render visible a low peak when in the original data it is preceded by a higher peak causing it to appear as a shoulder on the higher peak (Figures 2c and 2d).

### Comparison of the subtractive and carry-over correction methods

The automatic analyser response was digitised every second and the data stored in a PDP 11/70 computer. Both the subtractive and carry-over correction methods were implemented on the computer and performed on these data. The programs used were written by the authors in FORTRAN IV. To obtain the model peak for the subtractive method a standard specimen was analysed. In using the subtractive method it is necessary to choose a criterion for deciding the true height of a peak once it has been exposed, following the subtraction of the preceding peak. In this study the true height of a peak was taken as the value of the second to last data point on the peak apex (ie one second before the corresponding specimen was stopped being sampled). The automatic analyser was calibrated for both correction methods by using an internal standard.

A comparison of the subtractive and carry-over correction methods was performed as follows. A batch of 20 specimens was obtained by dividing ten plasma specimens into two portions. These specimens were then assayed in arbitrary order for their glucose content by an automatic analyser at a throughput of 78 samples/hour, which produced a corresponding series of 20 peaks (Figure 3). Another series of twenty peaks was obtained in a similar manner at a throughput of 150 samples/hour from another batch of ten different plasma specimens (Figure 4). (Those peaks corresponding to specimens prepared from the same plasma specimen have the same label in Figures 3 and 4).

Each correction method was assessed by its ability to correct to the same value the concentration of glucose in any

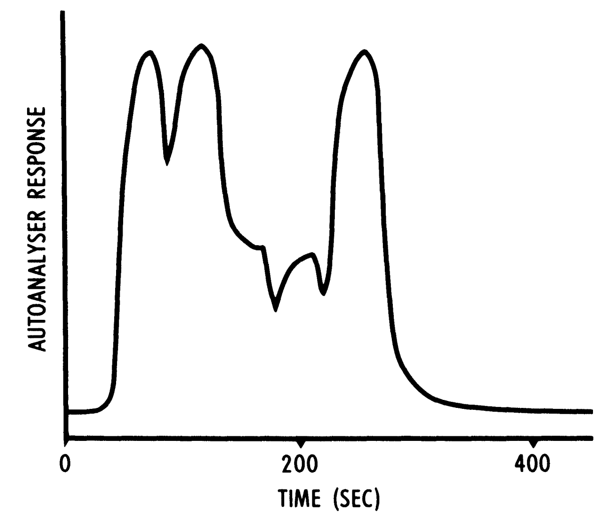

*Figure 2. (a) A series of five peaks produced by the analysis of five bicarbonate standards (each specimen was sampled for 40 sec, with wash solution being sampled between specimens for 6 sec). Note how the third peak appears as a shoulder on the trailing edge of the second peak.*

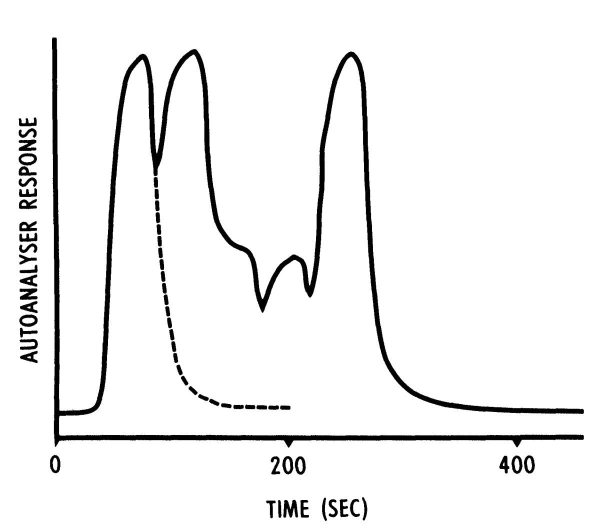

*(b) The trailing edge of the first peak (dotted line) is reconstructed by fitting the model peak (Figure 1) to the leading edge and apex of the first peak.*

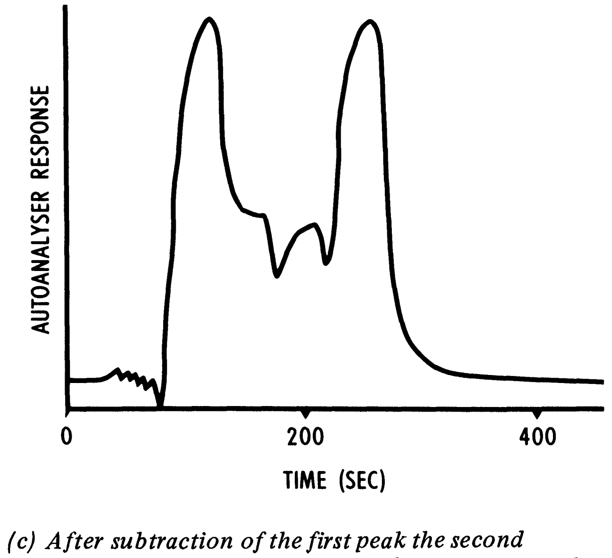

*(c) After subtraction of the first peak the second is exposed, so that its apparent height is its true height.*

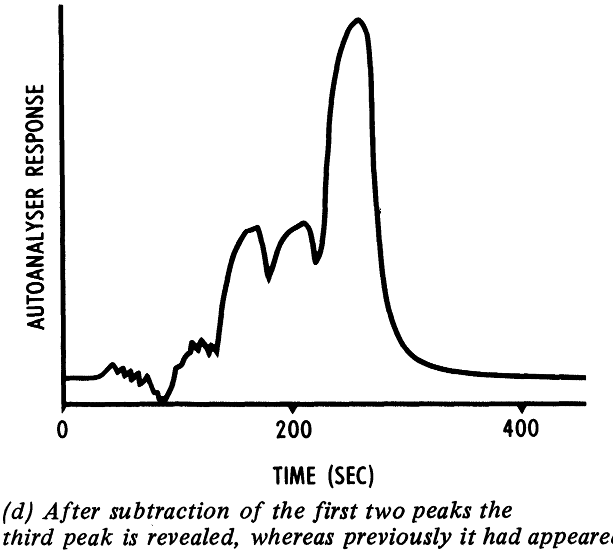

*(d) After subtraction of the first two peaks the third peak is revealed, whereas previously it had appeared as a shoulder on the second peak.*

pair of specimens prepared from the same plasma specimen (the glucose concentration being given by the corrected height of a peak). The corrected glucose concentrations, after analysis at throughputs of 78 samples/hour and 150 samples/hour, are given in Tables 1 and 2 respectively. The discrepancies between the glucose concentrations of corresponding specimens after correction by the two methods at both throughputs are given in Table 3, from which it can be seen that the subtractive and carry-over correction methods performed comparably at a throughput of 78 samples/hour. The mean discrepancy between any pair of corresponding specimens after correction is 0.14 mmol/l for the subtractive method and 0.18 mmol/l for the carry-over method. The respective maximum discrepancies are 0.40 mmol/l and 0.69 mmol/l. At a throughput of 150 samples/hour the mean discrepancies between the corrected heights of any pairs of corresponding peaks is 0.18 mmol/l for the subtractive method and 0.36 mml/l for the carry-over method. The respective maximum discrepancies are 0.41 mmol/l and 1.12 mmol/l. The subtractive correction method performs as well at a throughput of 150 samples/hour as at 78 samples/hour, whereas the carry-over correction method performs badly at the greater throughput (due to some of the smaller peaks appearing as poorly defined shoulders on preceding higher peaks - particularly the second of peaks L and M in Figure 4).

### Discussion

The study presented here compares the consistency with which the two correction methods will estimate the glucose concentrations of two specimens known to have the same concentrations. This is the most important feature required of a correction method, since any systematic error can be corrected for by suitable calibration. The results of the comparative study indicate that the subtractive method of automatic analyser peak height correction is capable of considerably increasing the throughput of the automatic analyser from that possible using the carry-over correction method. The method would be particularly useful for analyses in which there is a large range of expected analyte concentrations, so that many peaks would appear as shoulders.

Clearly, the subtractive correction method is limited by the accuracy with which each peak can be reconstructed by scaling the model peak. A possible improvement to the method as presented above would be to use a model peak obtained by averaging several isolated peaks, rather than using only one such peak. Also, there is inevitable contamination on the measured automatic analyser response (as evidenced by the ripples on the response peaks shown in Figures 1 and 2). Such contamination could adversely affect the determination of the true height of a peak, as performed in this study, after the preceding peak has been subtracted. Smoothing of the peak apex would alleviate the problem. The subtractive method is also limited by the digitisation of the automatic analyser response. A throughput of significantly greater than 150 samples/hour may be possible, but would require a greater data rate than one point/second.

**Table 1 The peak heights corresponding to the glucose concentrations of twenty plasma specimens after correction by the subtractive and carry-over correction methods. The throughput was 78 samples/hour (each specimen was sampled for 40 seconds, with wash solution sampled for six seconds between specimens).**

| Specimen label | Corrected glucose concentrations (millimole/litre) | |
|---|---|---|
| | Subtractive method | Carry-over method |
| A | 6.01 | 6.12 |
| B | 0.11 | 0.00 |
| C | 10.82 | 10.86 |
| D | 10,05 | 10.17 |
| E | 6.53 | 6.72 |
| F | 5.93 | 5.95 |
| G | 9.22 | 9.31 |
| H | 4.72 | 4.83 |
| I | 4.75 | 4.83 |
| J | 9.90 | 9.91 |
| E | 6.56 | 6.64 |
| I | 4.67 | 4.74 |
| D | 10.16 | 9.48 |
| H | 4.32 | 4.48 |
| C | 10.45 | 10.69 |
| G | 9.16 | 9.40 |
| B | 0.09 | 0.00 |
| F | 5.84 | 5.86 |
| A | 6.15 | 6.21 |
| J | 9.96 | 10.09 |

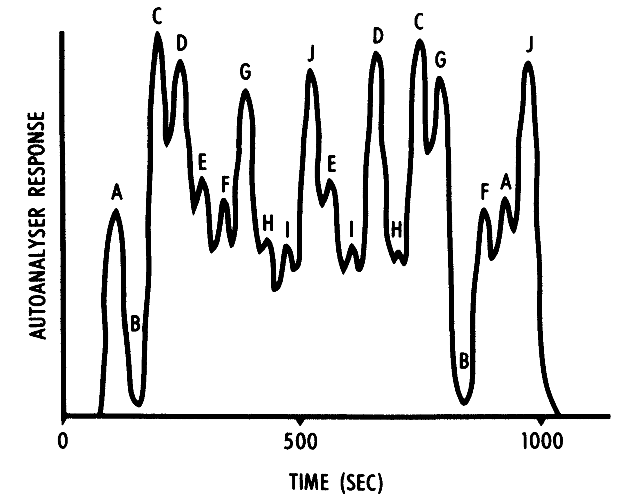

*Figure 3. Twenty peaks produced by the analysis of 20 plasma specimens for their glucose concentrations, at a throughput of 78 samples/hour (each specimen was sampled for 40 seconds with wash solution being sampled for 6 seconds between specimens). The peaks produced by corresponding specimens (i.e. those whose glucose concentrations are actually equal) have the same label.*

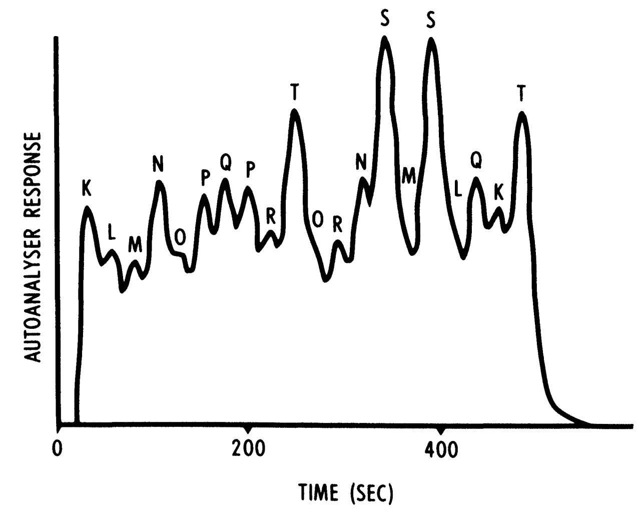

*Figure 4. Twenty glucose analysis peaks produced at a throughput of 150 samples/hour (each specimen was sampled for 20 seconds with wash solution being sampled for 4 seconds between specimens).*

**Table 2 The peak heights corresponding to the glucose concentrations of another batch of twenty plasma specimens after correction by the subtractive and carry-over correction methods. The throughput was 150 samples/hour (each specimen was sampled for 20 seconds, with wash solution being sampled for 4 seconds between specimens).**

| Specimen label | Corrected glucose concentrations (millimole/litre) | |
|---|---|---|
| | Subtractive method | Carry-over method |
| K | 6.36 | 6.12 |
| L | 5.11 | 4.89 |
| M | 4.78 | 4.64 |
| N | 7.72 | 7.72 |
| O | 4.92 | 4.76 |
| P | 6.94 | 6.92 |
| Q | 7.58 | 7.47 |
| P | 7.10 | 7.00 |
| R | 5.72 | 5.58 |
| T | 9.91 | 9.83 |
| O | 4.93 | 4.53 |
| R | 5.61 | 5.54 |
| N | 7.71 | 6.96 |
| S | 11.69 | 12.65 |
| M | 5.06 | 3.80 |
| S | 12.01 | 12.74 |
| L | 5.52 | 3.77 |
| Q | 7.26 | 7.68 |
| K | 6.50 | 6.22 |
| T | 9.91 | 10.00 |

The subtractive correction method can, with modest computer requirements, be adapted to on line data acquisition, and could be used in laboratories that are interested in a large throughput of specimens with minimal analytic error.

ACKNOWLEDGEMENTS

J. H. T. Bates wishes to acknowledge the financial support of the University Grants Committee of New Zealand. T. A. Walmsley acknowledges financial assistance from the Medical Research Council of New Zealand.

**Table 3 The discrepancies between the measured glucose concentrations of corresponding specimens after correction by the subtractive and carry-over methods at the two throughputs of 78 and 150 samples/hour.**

| Specimen label | Discrepancies between corresponding specimens (millimole/litre) | |
|---|---|---|
| | Subtractive method | Carry-over method |
| 78 samples/hour | | |
| A | 0.14 | 0.09 |
| B | 0.02 | 0.00 |
| C | 0.37 | 0.17 |
| D | 0.11 | 0.69* |
| E | 0.03 | 0.08 |
| F | 0.09 | 0.09 |
| G | 0.06 | 0.09 |
| H | 0.40 | 0.35 |
| I | 0.08 | 0.09 |
| J | 0.06 | 0.18 |
| Mean | 0.14 | 0.18 |
| 150 samples/hour | | |
| K | 0.14 | 0.10 |
| L | 0.41 | 1.12* |
| M | 0.28 | 0.84* |
| N | 0.01 | 0.76* |
| O | 0.01 | 0.23 |
| P | 0.16 | 0.08 |
| Q | 0.32 | 0.21 |
| R | 0.11 | 0.04 |
| S | 0.32 | 0.09 |
| T | 0.00 | 0.17 |
| Mean | 0.18 | 0.36 |

**Note the large differences produced by the carry-over method for those peaks that appear as shoulders on higher peaks.*

REFERENCES

[1] Bennet A., Gartelmann D., Mason J. I. and Owen J. A. (1970), *Clinica Chimica Acta*, **29**, 161-180.

[2] Abernathy M. H., Bentley G. T., Gartelmann D., Gray P., Owen J. A. and Quan Sing G. D. (1970), *Clinica Chimica Acta*, **30**, 463-482.

[3] Walker W. H. C. (1971), *Clinica Chimica Acta*, **32**, 305-306.

[4] Carlyle J. E., McLelland A.S. and Fleck A. (1973), *Clinica Chimica Acta*, **46**, 235-241.

[5] Crockford J. and Moore B. P. L. (1979), *Journal of Clinical Pathology*, **32**, 848-851.

